# Case Report: A liver metastasis 24 years after resection of a very low-risk jejunal gastrointestinal stromal tumor

**DOI:** 10.3389/fonc.2026.1773728

**Published:** 2026-04-13

**Authors:** Xiaonan Yin, Hongxin Yang, Luyao Sun, Yuan Yin, Bo Zhang

**Affiliations:** 1 Gastric Cancer Center, Department of General Surgery, West China Hospital, Sichuan University, Chengdu, Sichuan, China; 2 Department of Gastrointestinal Surgery, The Affiliated Hospital of Guizhou Medical University, Guiyang, Guizhou, China; 3 Department of Gastrointestinal Surgery, West China Hospital, Sichuan University, Chengdu, Sichuan, China

**Keywords:** gastrointestinal stromal tumor, imatinib, KIT mutation, late recurrence, liver metastasis, long-term follow-up

## Abstract

Gastrointestinal stromal tumors (GISTs) are the most common mesenchymal neoplasms of the gastrointestinal tract, with recurrence risk primarily determined by tumor size, mitotic index, site, and genetic profile. While most recurrences occur within 5 years post-resection, late recurrences (>10 years) are rare, posing diagnostic and therapeutic challenges. We report a unique case of hepatic metastasis from a jejunal GIST 24 years after initial curative resection of a very low-risk tumor (1.5 cm, ≤5 mitoses/50 HPF). The patient underwent jejunal GIST resection in November 2000, no KIT/PDGFRA mutational testing or adjuvant therapy was performed at the time. He remained recurrence-free for 24 years based on periodic abdominal imaging. In June 2024, abdominal ultrasound revealed a liver nodule (2.9×1.8 cm), confirmed by MRI/CT as a suspicious hepatic lesion. Laparoscopic right-sided complex hepatectomy was performed, and histopathology with immunohistochemistry (DOG1+/CD117+) and molecular testing (KIT exon 11 deletion) confirmed metastatic GIST. Adjuvant imatinib (400 mg/day) was initiated, with no recurrence at 18-month follow-up. This case challenges the assumption that very low-risk GISTs confer negligible long-term risk, highlighting their potential for extremely delayed metastasis. It underscores the need for lifelong vigilance, even in low-risk patients, and emphasizes the critical roles of advanced imaging, immunohistochemistry, and molecular diagnostics in diagnosing late metastases. Individualized follow-up strategies and consideration of long-term surveillance for selected low-risk GISTs warrant reevaluation in the era of molecular-targeted therapy.

## Background

Gastrointestinal stromal tumors (GISTs) represent the most common mesenchymal neoplasms of the gastrointestinal tract, originating predominantly from interstitial cells of Cajal lineage. These tumors are defined by gain-of-function mutations in the KIT or PDGFRA proto-oncogenes, which constitutively activate the KIT receptor tyrosine kinase signaling cascade, thereby driving neoplastic proliferation (1, 2). The risk of recurrence or metastasis in GIST is primarily determined by tumor size, mitotic index, site, and genetic mutation profile (3, 4). Conventionally, tumors with a size ≤ 5 cm and ≤ 5 mitoses/50 high-power fields (HPF) are considered very low or low risk, with a reported recurrence rate of <5%​ (5, 6). The liver and peritoneum are the most common sites of metastasis, while lung and bone metastases are rare (7, 8).

The majority of GIST recurrences occur within 5 years after surgery, late recurrences (>10 years post-resection) are rare, with only a small proportion of tumors recurring beyond the first decade (9). Cases of GIST recurrence after 20 or even 25 years​ have been rarely reported and often pose significant diagnostic and therapeutic challenges due to their indolent presentation and the long disease-free interval (10).

Adjuvant therapy with imatinib mesylate, a selective KIT and PDGFRA tyrosine kinase inhibitor, has significantly improved recurrence-free survival (PFS) and overall survival (OS) in high-risk GIST patients (11, 12). However, its role in low-risk or very-low-risk GISTs remains limited, and such patients have traditionally not received adjuvant treatment. The emergence of late metastases in these patients reinforces the need for individualized risk assessment and possibly long-term surveillance. Herein we report a case of liver metastasis 24 years after resection of very low-risk jejunal GIST.

## Case report

A 59-year-old man underwent surgical resection of a jejunal tumor (maximum diameter = 1.5 cm) in November 2000. Histopathological analysis of the resected specimen identified a neoplasm composed of spindle cells. Immunohistochemical evaluation demonstrated strong positivity for both DOG1 and CD117 (c-KIT), which are pathognomonic markers for GISTs. The final pathology report confirmed a diagnosis of a GIST, classified as very low risk for disease recurrence. At the time, KIT and PDGFRA mutational analyses were not performed, and the patient did not receive any therapy following surgery. He was subsequently managed with periodic abdominal contrast-enhanced CT scans, all of which showed no evidence of tumor recurrence over the next two decades.

**Figure 1:**
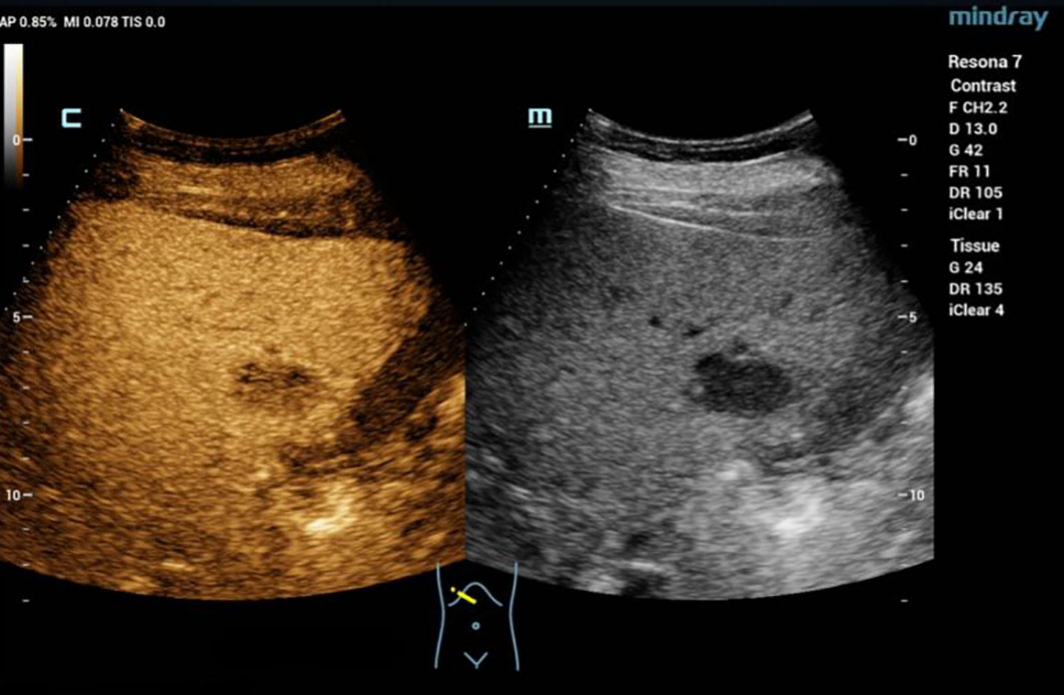
Abdominal ultrasonography revealed a hypoechoic mass in the right anterior and inferior segment of the liver, about 2.9 × 1.8 cm in size, with clear boundaries and relatively regular shape.

**Figure 2:**
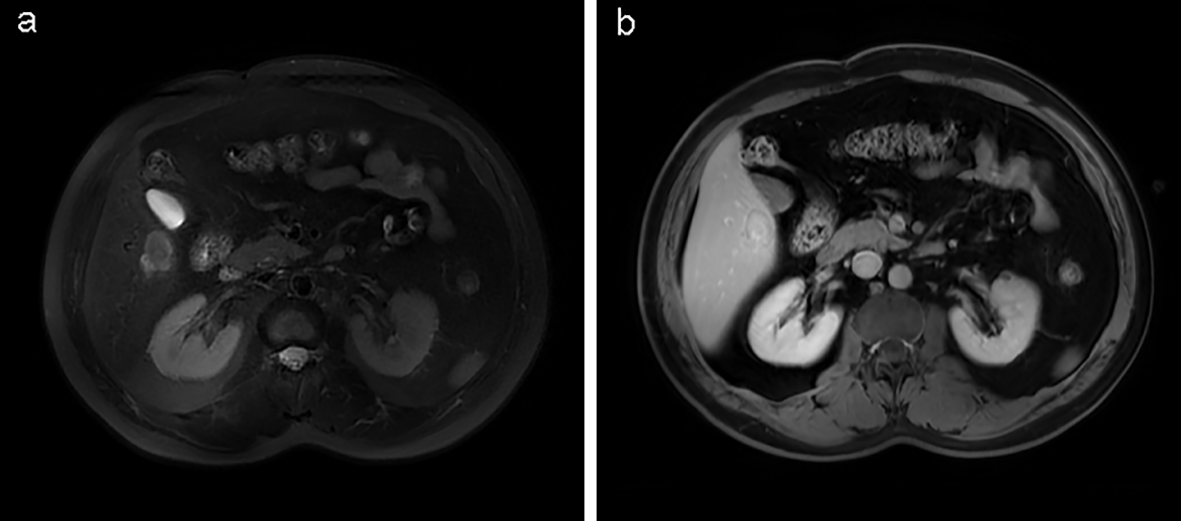
Liver MRI identified a lesion in the right posterior inferior hepatic segment, measuring approximately 2.2 × 3.1 cm. **(a)** T1-weighted imaging and **(b)** T2-weighted imaging revealed a lesion (white arrows) displaying slightly hypointense signal intensity on T1-weighted sequences and mixed hyperintensity on T2-weighted sequences.

**Figure 3:**
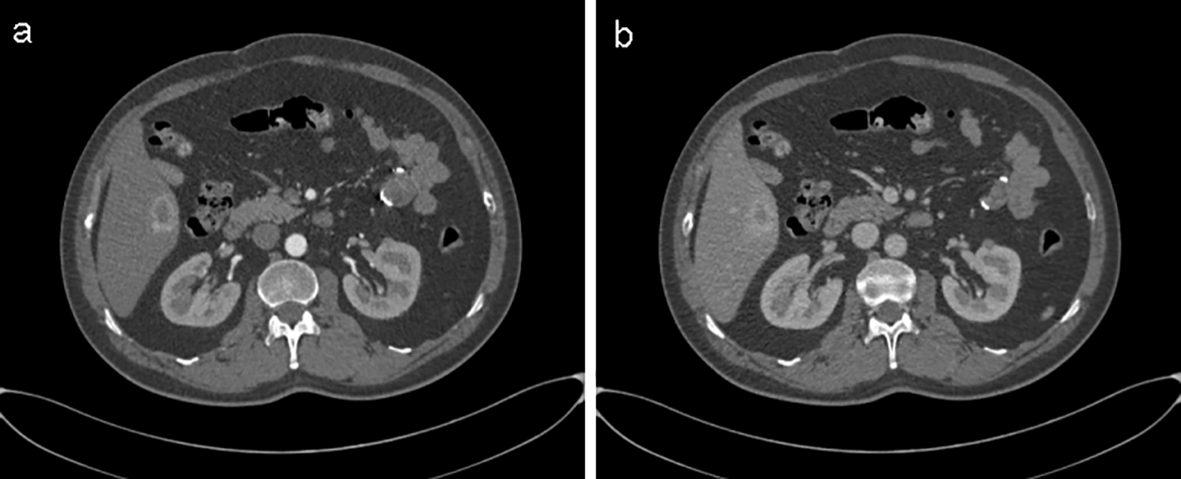
Abdominal contrast-enhanced CT revealed a hypodense hepatic nodule in the right posterior inferior lobe, measuring approximately 2.9 × 1.9 cm. **(a)** Arterial-phase imaging exhibited marked heterogeneous rim-like enhancement, while **(b)** portal venous-phase imaging demonstrated persistent enhancement, with a central area of comparatively diminished attenuation.

In June 2024, a routine abdominal ultrasound examination revealed a hypoechogenic mass, measuring approximately 2.9 × 1.8 cm, located in the right anterior inferior segment of the liver. The lesion exhibited well-defined margins and a relatively regular morphology, raising the suspicion of a primary or secondary hepatic malignancy (**Figure 1**). Subsequent liver magnetic resonance imaging (MRI) demonstrated a lesion measuring 2.2 × 3.1 cm, situated in the right posterior inferior hepatic segment. The lesion exhibited slightly hypointense signal on T1-weighted images and mixed hyperintensity on T2-weighted sequences, along with restricted diffusion. It showed marked heterogeneous rim-like enhancement in the arterial phase, diminished but persistent enhancement in the portal venous phase, and a slightly hypointense signal on the hepatobiliary phase. These imaging features were suggestive of a neoplastic lesion, chronic inflammatory or granulomatous lesions (**Figure 2**). Further evaluation with contrast-enhanced abdominal computed tomography (CT) identified a hypodense nodule, approximately 2.9 × 1.9 cm in size, in the right posterior inferior lobe of the liver. The nodule had ill-defined margins, with prominent heterogeneous ring-enhancement in the arterial phase and persistent enhancement in the portal venous phase, accompanied by a central area of relatively decreased attenuation (**Figure 3**). Concurrently, a comprehensive panel of serum tumor markers, including alpha-fetoprotein (AFP), carcinoembryonic antigen (CEA), cancer antigen 15-3 (CA15-3), carbohydrate antigen 19-9 (CA19-9), and cancer antigen 125 (CA125), was found to be within their respective normal reference ranges. Based on the radiological findings and unremarkable tumor marker profile, a preoperative diagnosis of a hepatic neoplasm was established, and the patient was subsequently scheduled for surgical resection.

**Figure 4:**
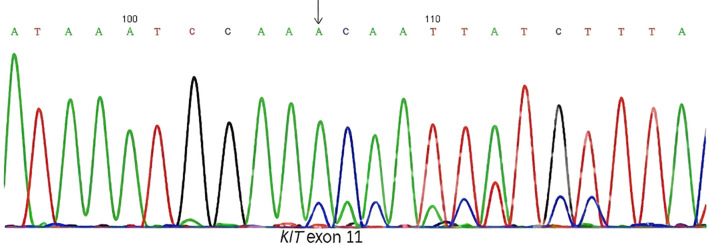
Molecular genetic analysis confirmed the presence of a deletion mutation in KIT exon 11 (p. Thr566_Pro573del), a canonical driver mutation commonly associated with GIST pathogenesis and therapeutic responsiveness to tyrosine kinase inhibitors.

On July 10, 2024, after completion of all necessary preoperative evaluations, the patient underwent laparoscopic right-sided complex hepatectomy (H67’). Postoperative histopathological examination of the resected specimen revealed a spindle cell tumor. Immunohistochemical staining was positive for DOG1 and CD117 (c-KIT), findings that were highly suggestive of a GIST. To confirm the diagnosis and guide further management, molecular genetic testing was performed, which identified a KIT exon 11 deletion mutation (**Figure 4**). This finding confirmed the diagnosis of a metastatic GIST, consistent with late hepatic metastasis from the previously resected jejunal GIST. The patient was started on adjuvant imatinib therapy at a standard dose of 400 mg daily. As of the latest follow-up, 18 months post-surgery, the patient remains clinically well with no evidence of recurrent or metastatic disease on imaging.

## Discussion

This case describes an extraordinary instance of late hepatic metastasis from a jejunal GIST, occurring 24 years after initial curative resection and originally classified as very low-risk. The most striking feature of this case is the remarkably long disease-free interval of 24 years before the development of a metastatic lesion, which challenges conventional assumptions regarding the natural history and timeline of GIST recurrence. A limited number of case reports have documented GIST recurrences occurring 20–30 years after the initial diagnosis, often involving the liver or peritoneum, and sometimes in patients whose primary tumors were classified as low-risk or even benign at the time of surgery (10, 13–15). These cases highlight the indolent yet persistent biological behavior of GIST, whereby residual tumor cells may remain clinically dormant for decades before eventual activation and metastatic progression.

From a diagnostic perspective, the hepatic lesion in our patient was initially detected on abdominal ultrasonography as a hypoechogenic mass. Its relatively well-defined borders and smooth, uniform margins initially raised suspicion for primary hepatocellular carcinoma, a common consideration in the evaluation of indeterminate liver lesions. This case highlights a significant clinical challenge in the diagnosis of late-presenting GIST metastases, particularly when recurrence occurs years after the initial resection of a small, low-risk, or molecularly uncharacterized primary tumor. In such scenarios, GIST is frequently omitted from the initial differential diagnosis. Subsequent imaging with MRI and CT revealed a constellation of findings, including heterogeneous rim-like arterial phase enhancement, persistent portal venous phase enhancement, restricted diffusion, and a hypointense signal in the hepatobiliary phase, all of which are features commonly associated with malignant hepatic lesions. While these imaging characteristics may overlap with those of hepatocellular carcinoma, cholangiocarcinoma, or other metastatic processes, they are also highly suggestive of GIST metastases, particularly those originating from the gastrointestinal tract (16, 17). The definitive diagnosis was ultimately established through histopathological examination of the resected liver lesion, which demonstrated a spindle-cell neoplasm. Immunohistochemical staining revealed strong and diffuse positivity for DOG1 and CD117 (c-KIT), markers that are highly specific for GIST. Molecular genetic analysis further identified a KIT exon 11 deletion mutation, the most prevalent and therapeutically significant mutation in GIST, strongly associated with responsiveness to imatinib mesylate (18).

Although primary hepatic GISTs are exceedingly rare and are seldom encountered in clinical practice, their possibility is generally entertained only after more common etiologies, particularly metastatic disease from a primary GIST of gastrointestinal origin, have been rigorously excluded. In the present case, multiple lines of evidence strongly favor a diagnosis of hepatic metastasis over a primary hepatic GIST. First, the patient has a well-documented history of a previously resected jejunal GIST, which was both histopathologically and molecularly confirmed to be a KIT-mutant tumor. Second, the hepatic lesion exhibited imaging features highly indicative of metastatic disease, including a hypodense appearance, irregular margins, and marked heterogeneous arterial phase enhancement with persistent portal venous phase uptake, findings classically associated with GIST metastases. Third, the absence of morphologic features typically seen in primary hepatic malignancies, such as vascular invasion or biliary communication, further supports a metastatic rather than a *de novo* hepatic origin. Finally, the patient’s clinical course, characterized by isolated hepatic progression in the setting of a known extrahepatic GIST, strongly suggests secondary hepatic involvement. Given the exceptional rarity of primary hepatic GISTs and the comprehensive clinical, radiologic, and pathologic evidence supporting a metastatic etiology in this case, we conclude that the most plausible and clinically coherent diagnosis is that of a hepatic metastasis from a jejunal GIST. This case underscores the critical role of immunohistochemistry and molecular genetic testing in establishing a definitive diagnosis of GIST, particularly in patients presenting with late metastatic disease or lesions that lack clear diagnostic features.

This case also carries significant implications for clinical practice, long-term patient management, and the design of follow-up strategies in GIST. First, it reinforces the concept that GISTs, even those originally classified as very low-risk or low-risk based on size and mitotic index, have the potential for extremely late recurrence, and that the risk may persist for decades. The traditional view that a long disease-free interval equates to cure or negligible future risk may not apply universally, especially in the context of molecularly driven neoplasms such as GIST. Second, it highlights the need for continued clinical vigilance and possibly lifelong follow-up in certain patients especially for patients with jejunoileal GISTs, even those with very-low or low-risk tumors, particularly in the modern era where molecular diagnostics and targeted therapies are readily available (13). Although adjuvant imatinib is not routinely recommended for low-risk GISTs, its use in the setting of late recurrence, especially when a driver mutation such as KIT exon 11 is present, has proven efficacy in controlling disease progression (8). In our case, the patient was started on adjuvant imatinib 400 mg daily following surgical resection of the metastatic lesion, and he remained free of recurrence at 18 months of follow-up, consistent with the known activity of imatinib in KIT-mutant GIST.

Furthermore, this case illustrates the diagnostic complexity that can arise when late metastases occur in atypical timeframes and anatomical locations. It serves as a reminder that metastatic GIST should be considered in the differential diagnosis of any new solid liver lesion in patients with a remote history of GIST, even in the absence of symptoms or risk factors. The overlapping imaging characteristics with other primary and secondary liver malignancies further emphasize the importance of a multidisciplinary approach, incorporating radiology, pathology, molecular diagnostics, and oncology, to ensure timely and accurate diagnosis.

## Conclusion

In conclusion, this case report describes an exceptional and instructive instance of hepatic metastasis from a jejunal GIST with very low risk occurring 24 years after initial resection, challenging conventional assumptions about the natural history and timeline of GIST recurrence. It highlights the need for lifelong clinical vigilance, even in patients with low-risk tumors and prolonged disease-free intervals.

The successful diagnosis and management of this case underscore the critical roles of advanced imaging, immunohistochemistry, and molecular testing​ in identifying late GIST metastases. Furthermore, it advocates for individualized follow-up strategies​ and raises the question of whether long-term surveillance should be considered in selected low-risk GIST patients, particularly in the modern era of molecular diagnostics and targeted therapy.

## Data Availability

The raw data supporting the conclusions of this article will be made available by the authors, without undue reservation.

## References

[1] Hirota S Isozaki K Moriyama Y Hashimoto K Nishida T Ishiguro S . Gain-of-function mutations of c-kit in human gastrointestinal stromal tumors. Science. (1998) 279:577–80. doi: 10.1126/science.279.5350.577, PMID: 9438854

[2] Heinrich MC Corless CL Duensing A McGreevey L Chen CJ Joseph N . PDGFRA activating mutations in gastrointestinal stromal tumors. Science. (2003) 299:708–10. doi: 10.1126/science.1079666, PMID: 12522257

[3] Liu XH Bai CG Xie Q Feng F Xu ZY Ma DL . Prognostic value of KIT mutation in gastrointestinal stromal tumors. World J Gastroenterol. (2005) 11:3948–52. doi: 10.3748/wjg.v11.i25.3948, PMID: 15991300 PMC4504903

[4] von Mehren M Joensuu H . Gastrointestinal stromal tumors. J Clin Oncol. (2018) 36:136–43. doi: 10.1200/jco.2017.74.9705, PMID: 29220298 PMC6553810

[5] Miettinen M Makhlouf H Sobin LH Lasota J . Gastrointestinal stromal tumors of the jejunum and ileum: a clinicopathologic, immunohistochemical, and molecular genetic study of 906 cases before imatinib with long-term follow-up. Am J Surg Pathol. (2006) 30:477–89. doi: 10.1097/00000478-200604000-00008, PMID: 16625094

[6] Miettinen M Sobin LH Lasota J . Gastrointestinal stromal tumors of the stomach: a clinicopathologic, immunohistochemical, and molecular genetic study of 1765 cases with long-term follow-up. Am J Surg Pathol. (2005) 29:52–68. doi: 10.1097/01.pas.0000146010.92933.de, PMID: 15613856

[7] Yu X Liang X Wen K . Clinical characteristics and prognosis of gastrointestinal stromal tumors with rare site metastasis (Review). Oncol Lett. (2022) 24:453. doi: 10.3892/ol.2022.13573, PMID: 36380879 PMC9650593

[8] Kelly CM Gutierrez Sainz L Chi P . The management of metastatic GIST: current standard and investigational therapeutics. J Hematol Oncol. (2021) 14:2. doi: 10.1186/s13045-020-01026-6, PMID: 33402214 PMC7786896

[9] Joensuu H Vehtari A Riihimäki J Nishida T Steigen SE Brabec P . Risk of recurrence of gastrointestinal stromal tumour after surgery: an analysis of pooled population-based cohorts. Lancet Oncol. (2012) 13:265–74. doi: 10.1016/s1470-2045(11)70299-6, PMID: 22153892

[10] Ginori A Scaramuzzino F Marsili S Tripodi S . Late hepatic metastasis from a duodenal gastrointestinal stromal tumor (29 years after surgery): report of a case and review of the literature. Int J Surg Pathol. (2015) 23:317–21. doi: 10.1177/1066896915573571, PMID: 25722316

[11] Dematteo RP Ballman KV Antonescu CR Maki RG Pisters PW Demetri GD . Adjuvant imatinib mesylate after resection of localised, primary gastrointestinal stromal tumour: a randomised, double-blind, placebo-controlled trial. Lancet. (2009) 373:1097–104. doi: 10.1016/s0140-6736(09)60500-6, PMID: 19303137 PMC2915459

[12] Joensuu H Eriksson M Sundby Hall K Hartmann JT Pink D Schütte J . One vs three years of adjuvant imatinib for operable gastrointestinal stromal tumor: a randomized trial. Jama. (2012) 307:1265–72. doi: 10.1001/jama.2012.347, PMID: 22453568

[13] Sun X Sun Y Xiong R Zhou Y Gao X Hou Y . Hidden risk factors for recurrence of jejunoileal Gastrointestinal stromal tumors with low NIH classification: implications for surveillance and adjuvant therapy. J Transl Med. (2025) 23:1156. doi: 10.1186/s12967-025-07163-1, PMID: 41121219 PMC12542470

[14] Kanda T Naito T Wakai A Iwafuchi Y Hirota S Ajioka Y . Late recurrence of low-risk gastrointestinal stromal tumor of jejunum diagnosed 30 years after tumor resection: A case report and literature review. Oncol Lett. (2023) 25:50. doi: 10.3892/ol.2022.13636, PMID: 36644139 PMC9811636

[15] Kikuchi H Yamamoto M Hiramatsu Y Baba M Ohta M Kamiya K . Manifestation of liver metastasis 13 years after gastrectomy for gastric GIST. Nihon Shokakibyo Gakkai Zasshi. (2006) 103:1055–60. 16953103

[16] Ruan J He Y Li Q Song M Jiang Z Mao K . CT feature of irregular extensive ulceration as a predictor of liver metastasis in gastric gastrointestinal stromal tumours. Eur Radiol. (2025) 35:2759–68. doi: 10.1007/s00330-024-11177-6, PMID: 39500800

[17] Mastoraki A Toliaki E Chrisovergi E Mastoraki S Papanikolaou IS Danias N . Metastatic liver disease associated with gastrointestinal stromal tumors: controversies in diagnostic and therapeutic approach. J Gastrointest Cancer. (2015) 46:237–42. doi: 10.1007/s12029-015-9748-6, PMID: 26163021

[18] Joensuu H Wardelmann E Eriksson M Reichardt A Hall KS Schütte J . KIT and PDGFRA mutations and survival of gastrointestinal stromal tumor patients treated with adjuvant imatinib in a randomized trial. Clin Cancer Res. (2023) 29:3313–9. doi: 10.1158/1078-0432.Ccr-22-3980, PMID: 37014660 PMC10472091

